# Dataset of usage pattern and energy analysis of an Internet of Things-enabled ceiling fan

**DOI:** 10.1016/j.dib.2023.108900

**Published:** 2023-01-13

**Authors:** Hashim Raza Khan, Muhammad Hashir bin Khalid, Urooj Alam, Mahnoor Atiq, Uvais Qidwai, Saad Ahmed Qazi

**Affiliations:** a NED University of Engineering and Technology, Karachi, Pakistan; b Qatar University, Qatar

**Keywords:** Internet of Things, Usage pattern, Appliance energy consumption

## Abstract

Many electrical appliances have progressed from sheer prototypes to viable products by being automated with the help of sensors and Internet of Things (IoT). In this data driven century, there aren't many data-centric solutions for the effective use of residential and commercial ceiling fans. For the said reason, sensors were installed on a remote-controlled BLDC ceiling fan, and a large amount of user data with environmental indicators such as temperature and humidity, was collected. This data along with the fan speed was logged to a cloud server over Wi-Fi using a Wi-Fi enabled microcontroller. The raw data consists of timestamp, temperature, humidity, and fan speed. The data is logged depending on the change of any parameter rather than a specific interval. The logged data is then visualized on the cloud server to monitor the usage patterns of the appliance and its subsequent energy consumption. The dataset is comprised of the fan data from the bedroom, living room, and lounge obtained by the resident's consent. This data is useful for data scientists, environmentalists, fan manufacturers, architects, social scientists, and several other field enthusiasts. The data can be analyzed based on monthly average temperature and humidity energy consumed, operational time per day or month and monthly/weekly summary of usage. Furthermore, by applying Artificial Intelligence (AI) algorithms on such data, it is feasible to extract patterns that indicate the appliance usage and identify changes in the daily routine. Many machine learning techniques can be applied on the dataset to introduce intelligent control of the appliance for adaptable operation without compromising on the comfort level of the user.

**Specifications Table**

**Table:** 

Subject	Computer Science and Energy Efficiency
Specific subject area	Internet of Things (IoT) and energy consumption pattern.
Type of data	The data is in the form of tables and datatype is numeric.
How the data were acquired	The data was acquired from several sensors retrofitted on a Brushless Direct Current (BLDC) ceiling fan namely, infrared (IR) receiver for controlling the fan speed from the IR remote, DHT11-Temperature and Humidity sensor, and DS3231 Real- Time Clock module. The data from these sensors was read using an ESP8266 Wi-Fi micro-controller module, which was then logged in flash files and sent to a central MySQL database server for storage and analysis.
	The derived variables calculated on the cloud from these measurements are: Operational time, energy consumed, and energy saved. The sensor-equipped ceiling fans were deployed at various typical residential locations with the consent of the residents.
Data format	Raw sensor values are represented as integers in the data. In addition, each data value has a date and time stamp that indicates when the value was recorded.
Description of data collection	The data was collected by installing the temperature, humidity, and infrared sensors on a ceiling fan. The circuit with the sensors and Wi-Fi-enabled microcontroller was interfaced with the driver circuit of the ceiling fan. The microcontroller was efficiently configured for IoT capabilities, including logging the data at dynamic intervals, i.e., when any variable changed. It efficiently managed the data transmission to a cloud server over the Wi-Fi network.
Data source location	City/Town/Region: Karachi Country: Pakistan
Data accessibility	Data of this article is available online at: Research Data-Dataset of usage pattern and energy analysis of an Internet ofThings-enabled ceiling fan

**Value of the Data**

• This dataset is useful for efficient monitoring and controlling the usage of the appliance [1]. The ineffective usage of the appliance can be monitored, and then corrective measures can be implemented to reduce the total energy consumption. The data can also be used to integrate past trend-based speed control in a ceiling fan to convert it into a fully automated appliance [2].
• This data is useful for data scientists, environmentalists, fan manufacturers, architects, social scientists, and a number of other field enthusiasts [3], [4], [5]. Environmentalists can use temperature and humidity data to investigate environmental changes, as well as see seasonal atmospheric variations and relate them to climate and weather. To make better design decisions, fan manufacturers may monitor regional statistics and observe the desired energy pattern of their appliance. Thermal comfort in interior spaces may be investigated by architects in order to design location and space profiles for building orientation plans that give the highest potential comfort for people.
• This dataset allows for significant data analytics and visualization, since the data may be studied based on monthly average temperature and humidity trend based on room types, weekly/monthly energy consumption for any room type, operating time of the appliance per day or month, and a monthly/weekly summary of usage.
• It is possible to extract trends that show the user's pattern and discover changes in the daily routine by using Artificial Intelligence (AI) algorithms on such data.
• Many machine learning techniques can be applied on the dataset to introduce intelligent control of the appliance for adaptable operation without compromising on the comfort level of the user [6,7].
• The data can be used by other researchers to carry out model validations, by providing a common benchmark for user's profile based on their usage.
• This data is also useful for developing AI based energy efficient appliances such as smart thermostats [8,9] which use machine learning algorithms to learn a household's heating and cooling patterns and adjust the temperature accordingly. This can help reduce energy consumption by up to 15%.
• Another example of use case of this data is in energy management systems [10,11] which use AI to analyze data from various sources (such as smart meters, weather forecasts, and building occupancy) and make recommendations for how to optimize energy usage. For example, a system might suggest turning off lights in an empty room or adjusting the thermostat when the building is unoccupied.

## 1 Objective

The objective of the study is to develop smart solutions on IoT-based fans for home and commercial users that have sensors to monitor the ambient room conditions in relation to user-preferred fan speeds. After data acquisition, the embedded machine learning models will enable the fan to learn the usage pattern and adjust the speed automatically, facilitating smart operation in a manually operated fan. Through this dataset, the usage pattern of fans in different rooms can be studied which can facilitate the development of smart features, such as automatic sleep mode in bedroom's fan for overnight fan speed regulation according to temperature, and slowing the fan speed at dinner time in the living room according to the trend. Moreover, the data can also aid in the research of the appliance operation based on seasonal and climate variations at various location or region. Different user profiles may be created to automate the appliance usage based on usage statistics and patterns.

## 2 Data Description

The IoT-enabled ceiling fan sent the collected data on the Digital Ocean server, where MySQL database was setup for storage. In order to make the dataset publicly available, data was extracted from the database and stored in three comma-separated values (csv) files. The dataset contains usage data of 3 fans of: bedroom, living room, and lounge, each as labelled in the csv file. Each file contains the columns of data received through the network into the appropriate fields including datetime, temperature, humidity, fan speed, operational time, energy consumed, and energy saved. Table 1 shows a generic view of the dataset. Each row in the dataset contains the information about a change in state of the indoor temperature, humidity, and fan speed as these are the raw values logged by the fan with the timestamps.

**Table 1:** Data description.

Column	Meaning	Unit	Example
datetime	The timestamp at which any variable changed (either temperature, humidity, or speed).	DD/MM/YYYY HH:MM:SS	07/02/2021 10:39
temperature	The ambient temperature read by the sensor.	°C	27
humidity	The ambient humidity read by the sensor.	%RH	35
speed	Fan speed which ranges from 0-powered off to 5-highest speed level. Each level represents a specific RPM value.	RPM or level number	2
opTime	Operational time-the time duration for which the fan operated at a specific speed.	seconds	90
eSpent	The energy consumed by the fan.	Joule or Ws	900
eSaved	The energy saved by the fan.	Joule or Ws	1260

While the data is not explicitly labeled with information about the season, the timestamp column contains the time with month and the year in which it was collected, readers can gain additional context by consulting a weather report or other source that provides information about the weather conditions during the time period in which the data was collected. A link to a relevant weather report is provided in [12].

Moreover, depending on the particular circumstances in which the data was acquired, there may or may not be a direct correlation between the indoor temperature and humidity sensor readings and the weather reports. In this case, the indoor temperature and humidity sensor readings are likely to be influenced by a number of factors, including the characteristics of the building or space in which the sensor is located (such as the presence of insulation, the size of the space, and the type of construction) as well as the activities occurring within the space (such as having dinner, cooking, and the use of appliances). As a result, the indoor temperature and humidity sensor values may differ somewhat from the weather readings. The weather readings, on the other hand, might give important context for evaluating the data by providing readers with a notion of the overall circumstances that may have impacted the readings.

For example, if the weather report indicates that the temperature outside was extremely cold or extremely hot during the time period when the data was obtained, readers might expect to observe some corresponding fluctuations in the indoor temperature and humidity values. Similarly, if the weather report indicates that there was a lot of precipitation (such as rain) during the time period in question, readers might expect some variations in the humidity levels.

From the dataset, the average of indoor temperature, humidity, and fan speed for each month of the year 2021 as given in the timestamp are calculated from Bedroom_Fan_1.csv as shown in Table 2. The average is calculated from the sum of all the rows in each of the corresponding columns. It can be interpreted from the Table 2 that which fan speed is preferable by the user according to the indoor temperature and humidity for each month. The same can be calculated for the other rooms and a macro trend can be observed.

**Table 2:** Monthly average of temperature, humidity and speed.

Month	Average of Temperature (°C)	Average of Humidity (%RH)	Average of Speed
January	21.14	60	1
March	31.41	63.03	1
April	33.13	56.18	1
June	33.99	73.88	4
July	32.17	74.77	3
August	31.62	79.67	2
September	35.49	72.86	5
October	32.63	78.21	2
December	23.81	68.25	1

**Fig. 1:**
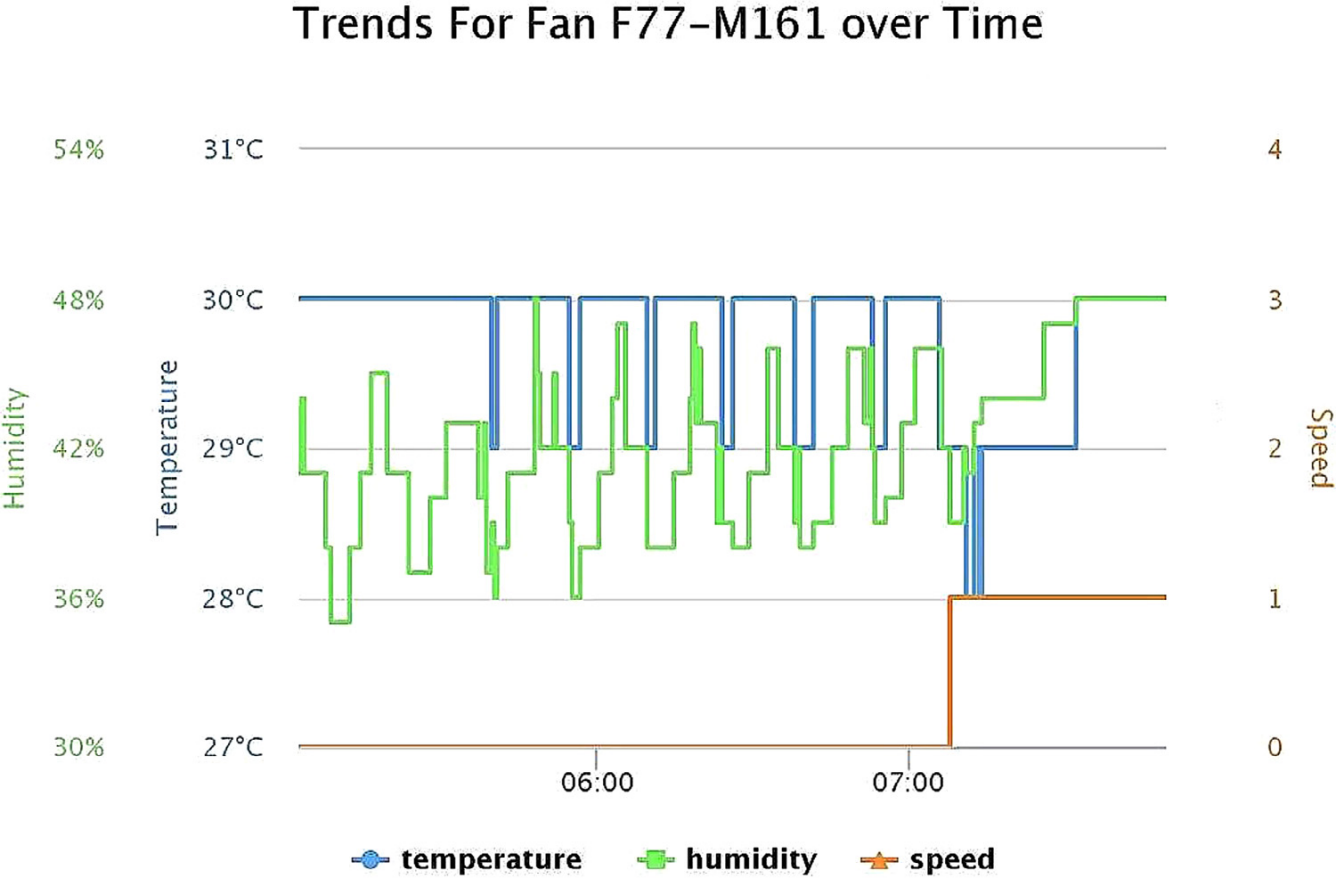
Fan usage trend over time.

**Fig. 2:**
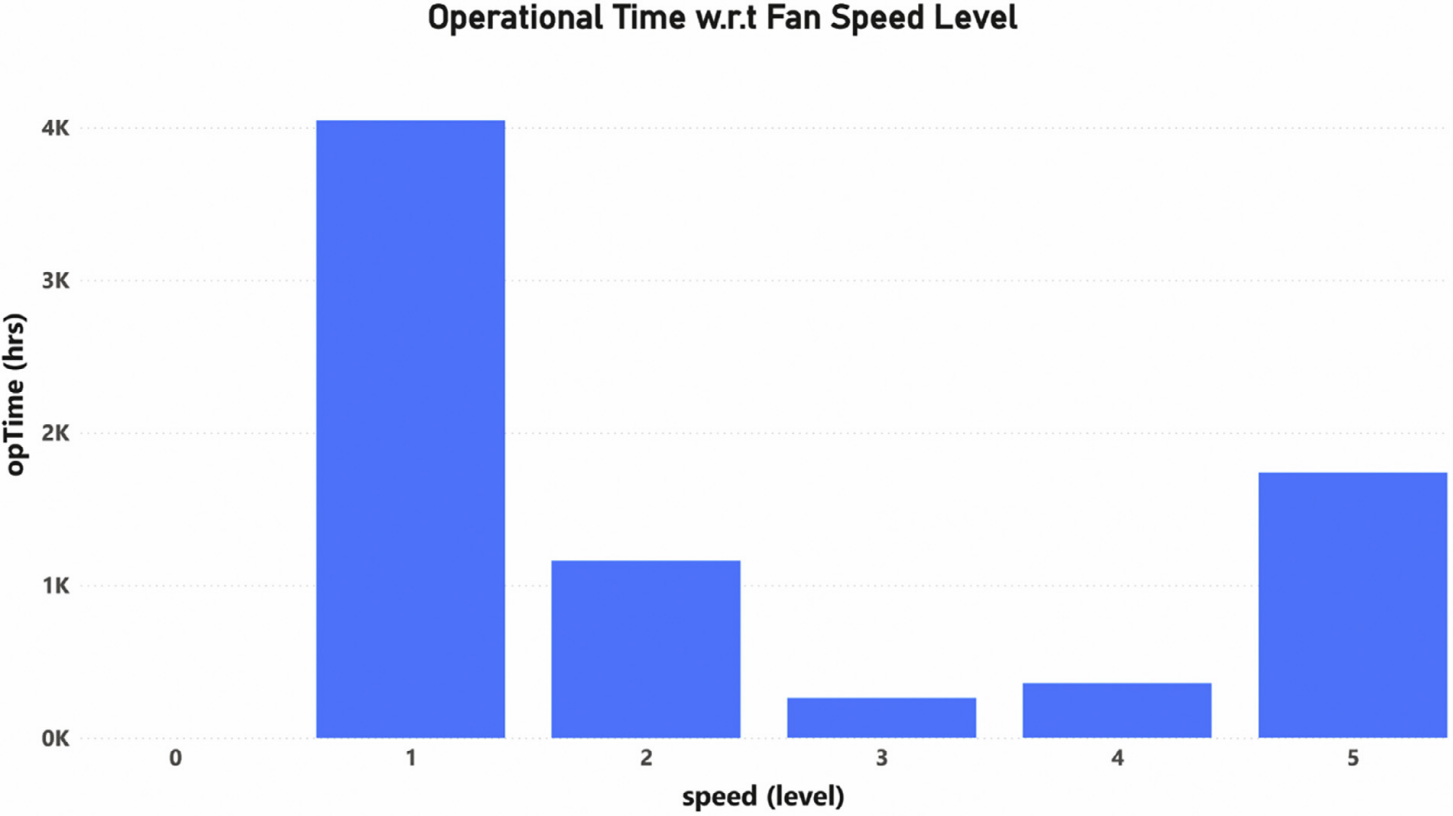
Operational time w.r.t fan speed per hour.

**Fig. 3:**
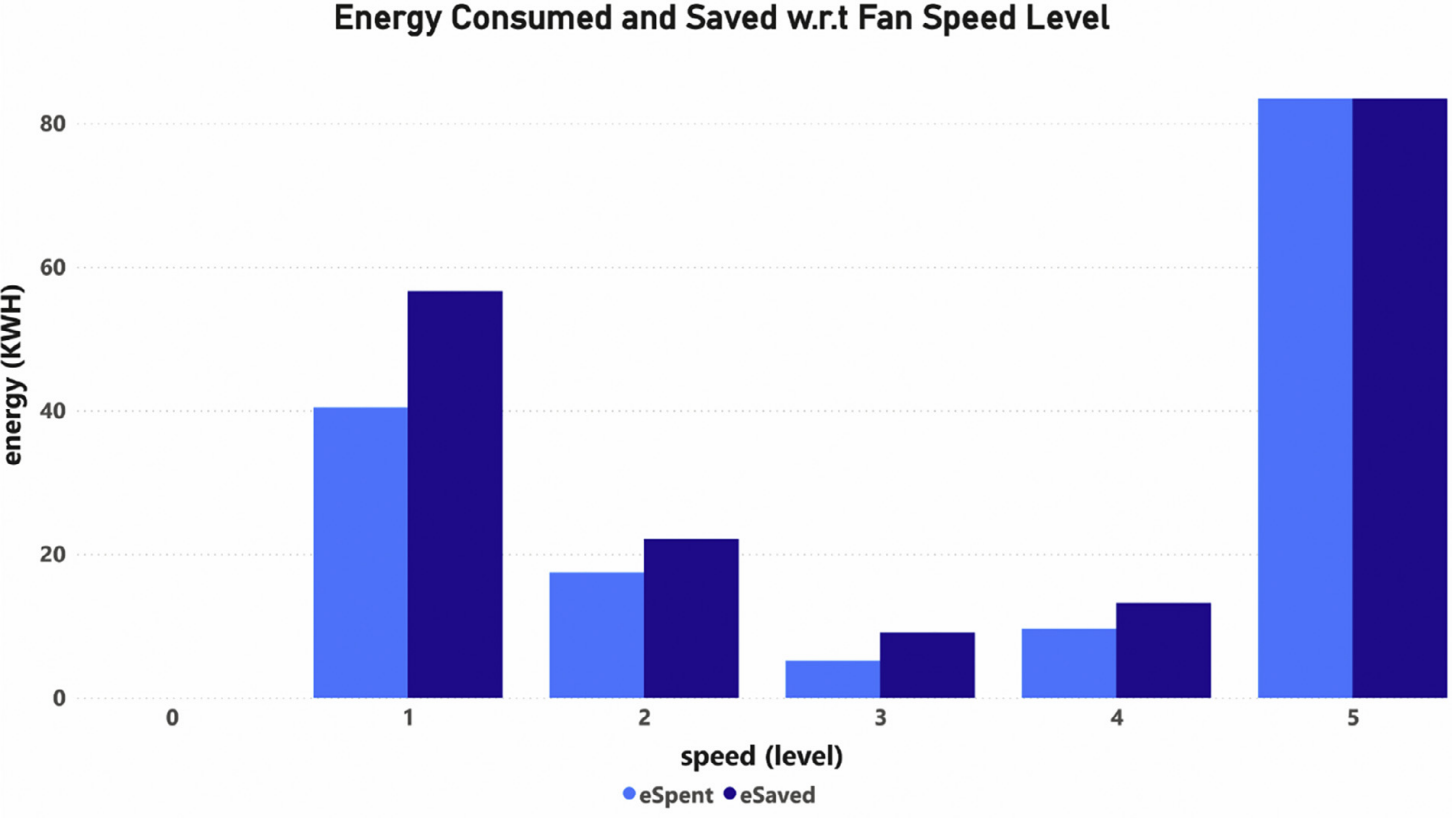
Energy consumed and saved w.r.t fan speed level.

The dataset can also be visualized to interpret more valuable insight such as Fig. 1, Fig. 2, Fig. 3. In Fig. 1, a small subset of the time series data of the fan speed is plotted along with the temperature and humidity using Highchart JavaScript library [13] to demonstrate an example of the usage trend from the raw data, showing how the consumption pattern may be studied. The remaining derived columns of operational time, energy consumed and saved were calculated on the cloud server. As mentioned in Table 1, the opTime is the time duration for which the fan operated at a specific speed level. The opTime value is calculated by subtracting the current timestamp (the time at which the speed change was logged) from the next timestamp (the time at which the next speed change was logged). This difference represents the total period of time that the fan operated at a certain speed level.as indicated in Eq. (1). By calculating the sum of the data column of opTime of Bedroom_Fan_1.csv, Fig. 2 provides an example of how many hours a fan has been operating at a particular speed.

(1) opTime=currentTimeStamp−nextTimeStamp

Furthermore, in order to determine the energy saved by the BLDC fans, power in Watts was measured at each speed level for both conventional AC and Brushless DC motor fans, using an electronic tachometer as shown in Table 3. The table shows the Revolution Per Minute (RPM) for the speed levels with corresponding value of power in Watts. Additionally, it can be seen that the BLDC fan uses 50% less power at the highest speed level, 5, than the conventional AC fan does. The same can be seen for other speed levels as well.

**Table 3:** Lookup table of power and RPM for fan speed levels.

Speed Level	BLDC Fan Power (Watts)	Conventional AC Fan Power (Watts)	Ps=Power Saved (Watts)	RPM
1	10	24	14	178
2	15	34	19	212
3	20	55	35	240
4	27	64	37	265
5	48	96	48	327

In the dataset, eSpent is computed from the product of BLDC fan power with the opTime as shown in Eq. (2) from [14]. The power saved, denoted as P_s_ (from Table 3), is determined from the difference between conventional fan power and BLDC fan power as shown in Eq. (3). Then, as shown in Eq. (4), eSaved is calculated using the product of P_s_ and operational time. The sum of data columns of eSpent and eSaved are plotted for each speed level in Fig. 3 as an example to show how much the energy is consumed and saved at each speed level for the Bedroom_Fan_1.csv.

(2) eSpent=BLDCFanPower(Watts)×opTime(sec)

(3) PowerSaved=Ps=ConventionalFanPower(Watts)−BLDCFanPower(Watts)

(4) eSaved=Ps×opTime(sec)

## 3 Experimental Design, Materials and Methods

The dataset addresses the problem of ineffective usage of the residential ceiling fan and to monitor the energy consumption. The designed hardware is thus deployed at a number of sites across the city with the permission of data sharing from the users. Residents went about their daily routines as usual and used the fan normally, resulting in reliable data acquisition.

**Fig. 4:**
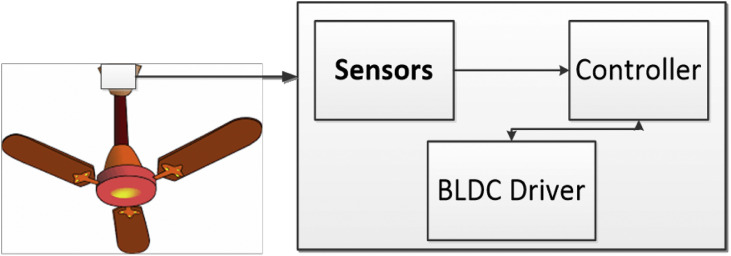
System on ceiling fan.

Based on Table 3, the energy efficient inverter technology of BLDC fans influenced the ceiling fan selection for the research. The hardware structure is illustrated in the Fig. 4. As indicated in the inset, the sensors, controller, and BLDC driving circuit were placed in the top fan canopy.

The BLDC driver was interfaced with the Wi-Fi enabled micro-controller through bypassing the IR sensor included in the DC fan. The on-board microcontroller and sensors were powered from the BLDC driver circuit's 15 volts DC supply by stepping it down to 5 volts using a buck converter. The micro-controller takes input from the infrared (IR) remote and controls the fan operations. With the onboard sensors, the temperature and humidity conditions along with the fan speed were logged in the on-flash memory of the microcontroller with the timestamp from the Real-Time Clock (RTC) module.

## 4 Materials

The hardware layout comprises of a control unit and a sensing unit. Each are explained briefly:

### 4.1 Control Unit

Control unit is based on NodeMCU which is an open-source software and hardware development environment that is built around a very inexpensive System-on-a-Chip (SoC) called the ESP8266. It is especially useful for IoT applications, thanks to its tiny footprint and built-in Wi-Fi support. To make the design feasible in terms of cost, we selected ESP8266 module version of NodeMCU with 4MB flash memory i.e., WeMos D1 mini.

### 4.2 Sensing Layer

Among the commonly used sensors in the sensing layer of IoT devices [15], DHT11- temperature and humidity sensor was finalized after effective testing and cost comparison of many locally available sensors. To store a timestamp with every data point, a Real-Time Clock (RTC) module comprising of DS3231 IC was also interfaced. An IR receiver was also interfaced to receive signals from the fan's IR remote to control the speed of the fan.

### 4.3 Method For Raw Data Acquisition

The microcontroller is programmed using Arduino IDE to read sensors and control the fan operation while also logging data. The control unit and sensing layer are then interfaced with the driver circuit of fan and placed in the top canopy of fan as illustrated in Fig. 4.

When the fan is operated, the microcontroller is configured with internet credentials provided by the user. It then checks for any change in status of sensor values, mainly temperature and humidity, along with the change in fan speed, then constructs a data string to store. The data string is stored in a JavaScript Object Notation (JSON) file in the on-flash memory of the microcontroller. The file is appended with data strings until it consumes 150 KB of flash memory, at which point it connects to the server through a POST request that includes its MAC address in order to be recognized by the server. The file is streamed using the Hypertext Transfer Protocol (HTTP). The POST API then sends the data to MySQL database set up on the cloud which stores and maintains the data. This data is further analyzed to monitor the usage trends of the fan.

Based on the response from the server at the device end, the sent file is removed from memory once it has been sent successfully. Meanwhile, another file is created to follow the same procedure thus providing reliable data for a long time.

## Ethics Statements

All ethical criteria, including obtaining consent from the residential owner, were followed prior to conducting the experiment.

## CRediT authorship contribution statement

**Hashim Raza Khan:** Conceptualization, Funding acquisition, Project administration, Resources, Supervision. **Muhammad Hashir bin Khalid:** Methodology, Resources, Investigation, Formal analysis, Data curation. **Urooj Alam:** Software, Validation, Investigation, Writing – original draft. **Mahnoor Atiq:** Data curation, Visualization, Writing – review & editing. **Uvais Qidwai:** Writing – review & editing. **Saad Ahmed Qazi:** Conceptualization, Funding acquisition, Resources.

## Declaration of Competing Interest

The authors declare that they have no known competing financial interests or personal relationships that could have appeared to influence the work reported in this paper.

## Data Availability

Research Data-Dataset of usage pattern and energy analysis of an Internet of Things-enabled ceiling fan (Original data) (Mendeley Data).
